# Effect of hydrophilic polymers on the solubility and dissolution enhancement of rivaroxaban/beta-cyclodextrin inclusion complexes

**DOI:** 10.1016/j.heliyon.2023.e19658

**Published:** 2023-09-01

**Authors:** Waqas Haider Khan, Sajid Asghar, Ikram Ullah Khan, Muhammad Irfan, Abdulrahman Alshammari, Muhammad Shahid Riaz Rajoka, Rabia Munir, Pervaiz A. Shah, Ikrima Khalid, Fizza Abdul Razzaq, Syed Haroon Khalid

**Affiliations:** a Department of Pharmaceutics, Faculty of Pharmaceutical Sciences, Government College University, Faisalabad, 38000, Pakistan; b Department of Pharmacology and Toxicology, College of Pharmacy, King Saud University, Post Box 2455, Riyadh, 11451, Saudi Arabia; c School of Dentistry, University of Maryland, Baltimore, MD, 21201, USA; d University College of Pharmacy, University of the Punjab, Lahore, 54590, Pakistan

**Keywords:** Rivaroxaban, βCD, Inclusion complex, Solubility, Soluplus

## Abstract

BCS class II drugs exhibit low aqueous solubility and high permeability. Such drugs often have an incomplete or erratic absorption profile. This study aimed to predict the effects of β-cyclodextrin (βCD) and different hydrophilic polymers (poloxamer 188 (PXM-188), polyvinyl pyrrolidone (PVP) and soluplus (SOLO)) on the saturated solubility and dissolution profile of hydrophobic model drug rivaroxaban (RIV). Binary inclusion complex with βCD were prepared by kneading and solvent evaporation method, at drug to cyclodextrin weight molar ratios of 1:1, 1:2, and 1:4. Saturated solubility of the hydrophobic model moiety was evaluated with βCD to explore the increment in saturated solubility. Dissolution test was carried out to assess the drug release from the produced binary inclusion complex in the aqueous medium. Solid state analysis was performed using Fourier transform infrared spectroscopy (FTIR), Differential scanning calorimetry (DSC), X-ray diffraction (XRD), and Scanning electron microscopy (SEM) techniques. When compared to pure drug, the binary complex (Drug: βCD at molar ratio of 1:2 w/w) demonstrated the best performance in terms of enhanced solubility and drug release. Furthermore, ternary inclusion complex was prepared with hydrophilic polymers SOLO, PVP K-30 and PXM-188 at 0.5%,1%,2.5%,5% and 10% *w/w* to optimized binary formulation RIV:βCD (1:2) prepared by kneading (KN) and solvent evaporation (S.E) method. The findings demonstrated that among ternary formulations (1:2 Drug: βCD: SOLO 10% S.E) manifested greatest improvement in saturated solubility and dissolution rate. Results of solubility enhancement and improvement in dissolution profile of model drug by ternary inclusion complexation were also supported by FTIR, DSC, XRD, and SEM analysis. So, it can be concluded that the ternary inclusion systems were more effective compared to the binary combinations in improving solubility as well as dissolution of hydrophobic model drug rivaroxaban.

## Graphical abstract

**Figure:**
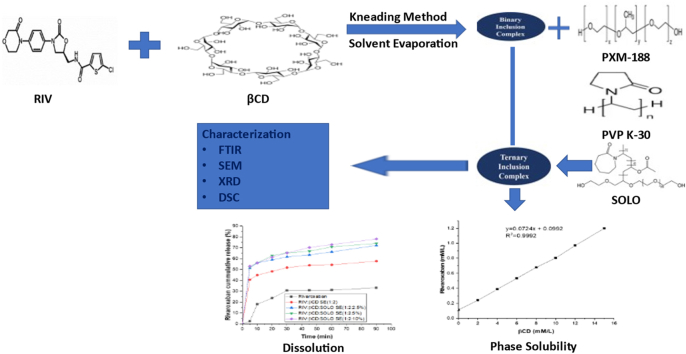

## 1 Introduction

Solubility is the most crucial part of new drug development process. Scientists and researchers have faced up hilled task to overcome the solubility related issues. It is estimated that about 40% of novel drugs or drugs those are currently under trails present solubility concerns [1]. BCS class II drugs exhibit low aqueous solubility and high permeability. Such drugs often have an incomplete or erratic absorption profile. For addressing the solubility issue in pharmaceutical product development, a number of approaches including solid dispersion, solvent deposition, micronization etc. Have been researched [2]. Each of these methods has merits and disadvantages of its own. Amongst these, Inclusion complexation is a classical way to ameliorate the solubility of a drug with natural cyclodextrins [2].

**Fig. 1:**
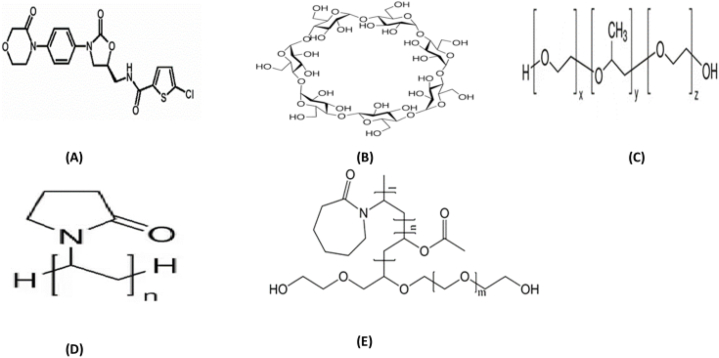
Chemical Structure of (A) Rivaroxaban, (B) βCD, (C) Poloxamer 188, (D) PVP K-30 and (E) Soluplus.

Cyclodextrins are a group of oligosaccharides, tend to capture the hydrophobic moiety into their cavity Fig. 1(B) and develop host-guest relationship [3]. Their solubilization efficiency is also enhanced in the presence of different hydrophilic polymers [4]. The extent of increment in the saturated solubility and dissolution rate of hydrophobic drugs differs depending on the polymers used as the carrier of the solid dispersion. Therefore, the selection of a polymer for inclusion complexation is important for the solubilization of poorly soluble drugs. PXM 188 Fig. 1(C) is used in this study due to attractive characteristics like emulsifying, solubilizing, and dispersing ingredients for pharmaceutical formulations [5].

Hydrophilic polymer such as polyvinylpyrrolidone (PVP K-30) has been widely used as carriers in pharmaceutical industry because of cost effectiveness, nontoxicity, biocompatibility and high solubilization efficiency. The approximate molecular weight of this polymer is 30,000 Da Fig. 1(D) [6]. Soluplus (SOLO) is another promising carrier for poorly water-soluble drugs. It is advanced polymeric solubilizer that has been utilized pharmaceutical industry to ameliorate the solubility of hydrophobic drugs like itraconazole, carbamazepine, griseofulvin and lovastatin [7]. SOLO is a graft amphipathic copolymer consists of polyvinyl caprolactam, polyvinyl acetate, and polyethylene glycol (13% PEG 6000, 57% vinyl caprolactam, and 30% vinyl acetate) as illustrated in Fig. 1(E) [8].

Novel Oral Anticoagulants (NOACs) as Rivaroxaban, Apixaban, Dabigatran and Edoxaban are used as alternative to vitamin K antagonists and lower molecular weight heparins (LMWHs). Rivaroxaban (RIV) an oxazolidinone derivative Fig. 1(A) and anticoagulant agent, is utilized for the prevention and cure of various arterial and venous metabolic disorders. It is generally regarded as safe and effective drug as compared to its competitors of vitamin K antagonists like Warfarin and Heparin [9]. It directly inhibits activation coagulation factor FXa and consequently prevents the formation and growth of clot. RIV does not require regular monitoring of dose and well suited for oral administration. Its predictable pharmacokinetics and pharmacodynamic characteristics make it a drug of choice in prevention and treatment of thrombosis after orthopaedic surgeries like knee or total hip replacement [10]. RIV is notorious for its low solubility and bioavailability. Its low solubility hampers its absorption and bioavailability of drug in gastro intestinal tract (GIT).

In previous works, RIV and βCD binary inclusion complex were prepared by different methods including spray drying and hot melt extrusion to enhance the solubility of RIV [11]. [12]. [13]. However, the percentage yield of spray-drying at laboratory scale with conventional spray-dryers is not optimal (20–70%) due to the loss of product in the walls of the drying chamber and the low capacity of the cyclone to separate fine particles [14]. Moreover hot melt extrusion is commercialized and expensive process which requires high input of energy [15]. In current investigation, binary and ternary inclusion complex formulations of RIV with βCD and different hydrophilic polymers were formulated by kneading and solvent evaporation techniques. Both of these methods were selected due to their simplicity, cost effectiveness and ease of scalability. Even though Numerous attempts have been made to enhance the solubility of rivaroxabin The current study differs from the previous work not only in terms of method selection but also in the selection of hydrophilic polymers which to the best of our knowledge have never been investigated for RIV solubility and dissolution enhancement before.

Thus, the current study aimed to predict the effects of βCD and different hydrophilic polymers (PXM-188, PVP K-30 and SOLO) on the saturated solubility and dissolution profile of hydrophobic model drug rivaroxaban. In this respect, binary inclusion complex of RIV were prepared with βCD first at different molar ratios of 1:1, 1:2, and 1:4 followed by preparation of ternary inclusion complexes of RIV with different hydrophilic polymers (PXM-188, PVP K-30 and SOLO) at different molar ratios of 0.5%,1%, 2.5%, 5.0% and 10.0% w/w of binary inclusion complexes. By using FTIR, SEM, XRD, and DSC techniques, the solid-state properties of binary and ternary inclusion complexes were studied and the effect of selected polymers on the saturation solubility and dissolution profile of RIV was evaluated.

## 2 Materials and methods

### 2.1 Materials

Rivaroxaban (RIV) was received as a gift sample from Consolidated Chemical Laboratories Lahore, (Pvt) Ltd, Pakistan. β-Cyclodextrin (βCD) was also a kind gift from Roquette (Lestrem, France). Soluplus (SOLO), Poloxamer 188 (PXM-188) and Polyvinyl pyrolidine (PVP K-30) were bought from Sigma-Aldrich (St. Louis, MO, USA). All other chemicals and solvents employed in this study were of analytical grade.

### 2.2 Methods

#### 2.2.1 Phase solubility

Compatibility of host and guest relationship was determined by Higuchi-Connors phase solubility method [16]. Excess amount of drug was added in flask containing different molar ratios of βCD mixed in distilled water. Flasks were placed in water bath (TSSWB15-USA) at 37 ± 2 °C with speed of 100 rpm for 72 h. Centrifugation of the saturated solutions were done at 6000 rpm for 30 min. Supernatant were filtered with 0.45 μm syringe filter and suitable dilutions were made to estimate the phase solubility in UV Spectrophotometer (CECIL 7400-S, Cambridge, UK**)** in triplicate manner at 248 nm. Stability constants (K_S_) and complexation efficiency (C.E) were calculated from the phase solubility diagram using equations (1.1) and (1).2 respectively.

(1.1) $$Stability\;constant\;\left(Ks\right)=\frac{slope}{So\left(1-slope\right)}$$

in this equation S_o_ is considered as saturated solubility of RIV in pure water; whereas value of slope is calculated by plotting RIV concentration against concentration of βCD.

(1.2) $$Complexation\;efficiency\;\left(C.E\right)=\frac{slope}{1-slope}$$

Gibbs free Energy can also be used as indicator for complexation process, which can be calculated from equation (1.3) stated below

(1.3) ΔG°=−2.303RTlog(So/Ss)

whereas, So/Ss is the ratio of the molar solubility of RIV in aqueous solution of cyclodextrin to that of the pure water.

#### 2.2.2 Preparation of binary inclusion complexation

Binary inclusion complex of RIV with βCD were formulated in various weight molar ratios of 1:1, 1:2, and 1:4. In kneading method, RIV and β-CD were kneaded together in mortar for 45 min, with equal amounts of water: ethanol (1:1) and dried at room temperature for 24 h. Kneaded mass was sieved through sieve number 60 [17].

For solvent evaporation method, the aqueous phase containing βCD and organic phase having RIV dissolved in ethanol were thoroughly mixed at magnetic stirrer. Electric oven was used to evaporate the solvent at 50 °C for 24 h. Sieving was done prior to its storage [17].

#### 2.2.3 Formulation of ternary complexes of RIV

Ternary complex of RIV were prepared with different hydrophilic polymers like PXM-188, PVP K-30 and SOLO at different molar ratios 0.5%,1%2.5%, 5.0% and 10.0% w/w of binary inclusion complexes by suitable methods [18].

#### 2.2.4 Solubility studies

Classical shake flask method was used to carry out the saturated solubility estimation of binary inclusion complexes of drug [19]. Accurately measured amount of inclusion complex was added in vials containing 5 mL of water, after vortex for sufficient time they were immersed in shaking water bath ‘‘TSSWB15-USA” for 72 h. After centrifugation at 6000 rpm for 30 min, membrane filters were used to filter the supernatant. Absorbance of the samples was determined in UV spectrophotometer ‘‘CECIL 7400-S, Cambridge, UK**”** after proper dilutions at 248 nm in triplicate manner.

#### 2.2.5 *In Vitro* Dissolution studies

Dissolution is a critical factor in preformulation studies, which affects the bioavailability of the moiety. *In vitro* dissolution studies of pure drug and inclusion complexes were performed using the paddle apparatus or USP dissolution apparatus type II (PTWS 3CE, Pharma test, Hainburg, Germany) [20]. Dissolution media consisting of 900 mL of distilled water at 37.0 ± 0.5 °C and paddle speed of 100 rpm was added to each vessel followed by incorporation of formulations equivalent to 10 miligram of drug susbsequently. Different samples were taken at pre-determined time intermissions of 0.5, 10, 20, 30, 45, 60 and 90 min. Absorbance of samples were measured in triplicate manner after filtration.

Dissolution efficiency at 60 min (DE_60_ min), expressed as “the area under the dissolution curve up to 60 min, expressed as a percentage of the area of the rectangle described by 100% dissolution in the same time” [21]. It can be calculated by using equation (1.4) mentioned below

(1.4) $$DE\left(\%\right)=\frac{\int_{0}^{t}y\;X\;dt}{y\;100\;X\;t}\;X\;100\%$$

in the above-mentioned equation y is the percentage of dissolve quantity of RIV. It was critical to evaluate the performance of variety of carriers in inclusion complexation.

#### 2.2.6 Fourier transform infrared spectroscopy (FTIR)

In FTIR (BRUKER Tensor II-Alpha, Berlin, Germany), RIV, binary and ternary inclusion complex samples were scanned over 400 to 4000 cm^−1^ spectral range. Potassium bromide (KBr) disk method was used to obtained FTIR spectrum of RIV and complexes.

#### 2.2.7 Scanning electron microscopy (SEM)

Surface morphology of pure drug and complexes were elucidated with the help of Scanning electron microscope (JSM-6480, Tokyo, Japan). Tested samples were thinly coated by gold in order to improve the electrical conductivity of prepared formulations prior to SEM testing.

#### 2.2.8 X-ray diffractometry (XRD)

X-ray Diffractometry was employed to evaluate the physical state of all samples. The X-ray tube was derived at 30 mA current and potential difference of 30 kV. Samples were scanned from 10° to 50° in the range of 2θ, at 0.02°/s increment.

#### 2.2.9 Differential scanning calorimetry (DSC)

Thermal analysis of pure RIV and inclusion complexes were studied using Differential scanning calorimetery technique (Universal V4.2 E TA Instruments, Newcastle, USA). 5 mg sample was placed in differential scanning calorimetry aluminium pan and heated in presence nitrogen (20 mL/min) at a rate of 10 °C/min over a thermal range between (30–300 °C).

### 2.3 Statistical analysis

All statistical analysis were performed using SPSS (version 22) and Quantitative results were analyzed and depicted as mean ± standard deviation (SD). One way analysis of variance (ANOVA) was employed to analyze the solubility and dissolution profile of binary and ternary inclusion complexes. When there was a statistically significant difference, a post hoc Tukey HSD (Honestly significant difference) test was performed. A statistically significant difference was considered at p < 0.05.

## 3 Results and discussion

### 3.1 Phase solubility

**Fig. 2:**
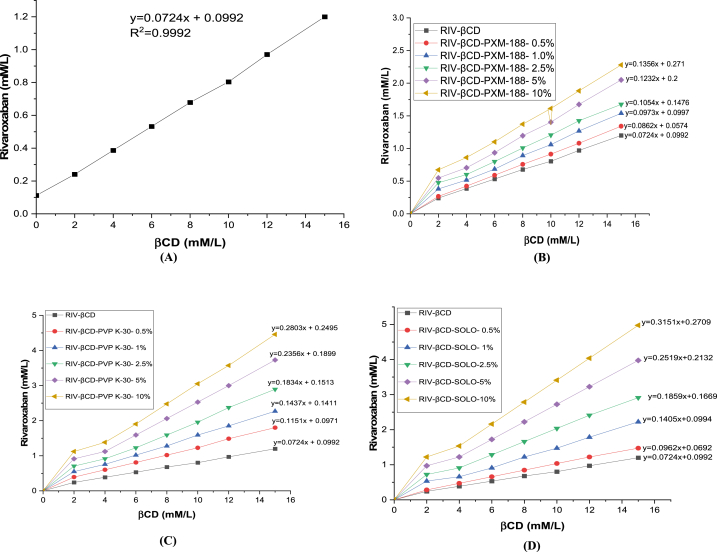
RIV phase solubility diagram in (A) βCD aqueous solution. (B) βCD aqueous solution with and without PXM-188. (C) βCD aqueous solution with and without PVP K-30. (D) βCD aqueous solution with and without SOLO.

Phase solubility curve of βCD and RIV was depicted in Fig. 2 (A). Phase solubility result revealed that increase in concentration of βCD contributed to increase in the solubility of RIV. This might be due to the solubilization efficiency and cavity dimensions of the βCD [22]. It could be classified into the AL type solubility diagram category.

The incorporation of hydrophilic polymers also influenced positive effects to ameliorate the solubility of binary complexes. SOLO Fig. 2(D) depicted the maximum value of increase in solubility at the same concentration of other polymers closely followed by PVP K-30 Fig. 2 (C) and PXM-188 Fig. 2 (B). This could be justified by the chemical nature of polymer and formation of micelles like structure with the active moiety [23]. The stability constant values of complexes were ranged in the following order: RIV: βCD: SOLO (0.5%,1%,2.5%,5% and 10%) > RIV: βCD: PVP K-30 (0.5%,1%,2.5%,5% and 10%) > RIV: βCD: PXM-18 (0.5%,1%,2.5%,5% and 10%) > RIV: βCD, depicting the higher affinity of SOLO for the studied model drug as compared to other hydrophilic polymers and βCD. Small stability constant value for binary complex indicated partial entrapment, on the other hand highest value indicated the maximum drug release from the ternary complex (reported values 200 - 5000 M^−1^) [24]. The higher values of stability constant were also instrumental in determining the existence of strong interaction between RIV and inclusion derivatives [25]. Complexation efficiency is also a crucial parameter in evaluating the solubility of inclusion complexes. The values of stability constant and complexation efficiency are shown in Table 1**.**

**Table 1:** Complexation efficiency and stability constant values of inclusion complexes.

Inclusion Complexes	Stability Constant (M^−1^)	Complexation Efficiency
RIV: βCD	699.09	0.078
RIV: βCD: PXM-188 (0.5%)	864.2	0.096
RIV: βCD: PXM-188 (1%)	1221.4	0.136
RIV: βCD: PXM-188 (2.5%)	1399.1	0.156
RIV: βCD: PXM-188 (5%)	1922.3	0.214
RIV: βCD: PXM-188 (10%)	2378.1	0.265
RIV: βCD: PVP K-30 (0.5%)	1081.1	0.120
RIV: βCD: PVP K-30 (1%)	1487.2	0.165
RIV: βCD: PVP K-30 (2.5%)	2096.9	0.245
RIV: βCD: PVP K-30 (5%)	3371.9	0.376
RIV: βCD: PVP K-30 (10%)	3769.6	0.420
RIV: βCD: SOLO (0.5%)	2359.4	0.263
RIV: βCD: SOLO (1%)	3046.4	0.340
RIV: βCD: SOLO (2.5%)	3400.8	0.379
RIV: βCD: SOLO (5%)	4139.9	0.458
RIV: βCD: SOLO (10%)	4759.6	0.536

Gibbs free energy could also be determined from phase solubility diagram. The values of Gibbs free energy were critical to understand transfer process of RIV from pure water to aqueous solution of βCD. The values were all negative for βCD at various concentrations as sown in Table 2, thus hinting the spontaneous nature of RIV solubilization. These values decreased with increasing concentration of βCD, thereby demonstrating that the reaction became more favourable as the concentration of βCD increased [26].

**Table 2:** RIV: CD complex's Gibbs free energy in water at 25 ± 2 °C.

βCD (mM/L)	ΔG^0^ (kcal mol^−1^)
2	−0.454
4	- 0.734
6	- 0.924
8	- 1.068
10	- 1.168
12	- 1.279
15	- 1.405

### 3.2 Solubility study

RIV is practically insoluble in aqueous media having solubility of 5.11 μg/mL in distilled water. Solubility of RIV was enhanced by binary inclusion complexes prepared by different methods as shown in Table 3. Data indicate that all techniques, though to various degrees, could improve RIV's solubility. The most significant increase in RIV's solubility with βCD was obtained by the kneading method, which was followed by solvent evaporation (P < 0.05). At a molar ratio of 1:2, the solubility of RIV/βCD was 42.21 μg/mL, which was 8.26 times more than that of the pure medication. RIV solubility, however, did not statistically significantly increase with the further increase in βCD quantity from 1:2 to 1:4 (P > 0.05). The kneading procedure, which produced the highest RIV solubility at comparable molar ratios for βCD, was the best of the two preparation methods. RIV solubility did not, however, statistically significantly increase with the further increase in βCD quantity from 1:2 to 1:4 (P > 0.05). Sherje et al. (2018) also prepared the binary complex of RIV by different methods and concluded that method of preparation had a pronounced effect on the solubility enhancement of RIV [19]. Based on the aforementioned information, inclusion complexes made using the 1:2 kneading and solvent evaporation method were optimized for ternary complexation.

**Table 3:** Saturated Solubility values for RIV, binary and ternary inclusion complex. Mean ± SD, n = 3.

Methods of Preparation	RIV: βCD: polymer (w/w)	Solubility ug/mL
	RIV: βCD (1:0)	5.11 ± 0.03
Kneading Method	RIV: βCD KM (1:1)	40.76 ± 0.03
	RIV: βCD KM (1:2)	46.20 ± 0.1
	RIV: βCD KM (1:4)	42.67 ± 0.07
	RIV: βCD:PXM 188 KM (1:2:2.5%)	60.31 ± 0.2
	RIV: βCD: PXM 188 KM (1:2:5%)	65.32 ± 0.27
	RIV: βCD:PXM 188 KM (1:2:10%)	71.58 ± 0.21
	RIV: βCD:PVP K-30 KM (1:2:2.5%)	78.88 ± 0.38
	RIV: βCD: PVP K-30 KM (1:2:5%)	81.51 ± 0.46
	RIV: βCD:PVP K-30 KM (1:2:10%)	84.76 ± 0.22
	RIV: βCD:SOLO KM (1:2:2.5%)	190.55 ± 0.37
	RIV: βCD:SOLO KM (1:2:5%)	220.60 ± 0.21
	RIV: βCD:SOLO KM (1:2:10%)	242.84 ± 0.13
Solvent Evaporation	RIV: βCDSE (1:1)	38.87 ± 0.61
	RIV: βCD SE (1:2)	42.21 ± 0.41
	RIV: βCD SE (1:4)	40.41 ± 0.15
	RIV: βCD:PXM 188 SE (1:2:2.5%)	90.18 ± 0.16
	RIV: βCD:PXM 188 SE (1:2:5%)	96.60 ± 0.28
	RIV: βCD: PXM 188 SE (1:2:10%)	102.71 ± 0.16
	1:2 (RIV: βCD: PVP K-30 SE (1:2:2.5%)	96.09 ± 0.78
	RIV: βCD: PVP K-30 SE (1:2:5%)	100.23 ± 0.33
	RIV: βCD: PVP K-30 SE (1:2:10%)	105.09 ± 0.2
	RIV: βCD: SOLO SE (1:2:2.5%)	247.48 ± 0.24
	RIV: βCD: SOLO SE (1:2:5%)	263.19 ± 0.51
	RIV: βCD: SOLO SE (1:2:10%)	281.27 ± 0.4

Ternary complexes depicted superior results than binary complexes largely due to the presence of hydrophilic polymers. PVP K-30 showed improved solubility results than PXM- 188. This could be credited to the less crystalline structure of inclusion mixtures as PVP K-30 has the tendency to inhibit the crystallization [27].

SOLO radically enhanced the solubility of RIV from 5.11 μg/mL to 281.27 μg/mL prepared by solvent evaporation method. This was mainly due to the chemical nature of the polymer and tendency to form micelles like structure around the active molecule. Lee et al. (2021) in his work also reported that SOLO had ameliorated the solubility of RIV in novel hot-melt extruded solid dispersion method [12]. Hence, it can be concluded that SOLO is a polymer of choice in enhancing the solubility of RIV due to its chemical structure.

### 3.3 In Vitro Dissolution studies

**Fig. 3:**
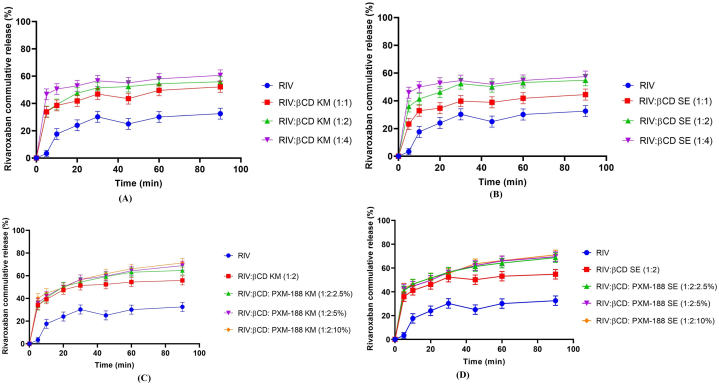

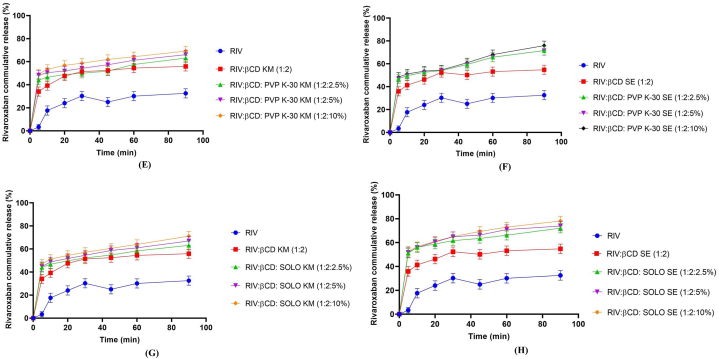
In Vitro Dissolution studies of Inclusion complexes (A) RIV-βCD KM B) RIV-βCD SE, (C) RIV:βCD: PXM-188 KM (D)) RIV:βCD:PXM-188 SE (E) RIV:βCD:PVP K-30 kM F) RIV:βCD:PVP K-30 SE (G) RIV:βCD:SOLO KM (H) RIV:βCD:SOLO SE. Mean ± SD, n = 3.

Cumulative dissolution release profile of pure RIV, binary complexes and ternary inclusion complexes prepared by different methods are shown in Fig. 3(A-H). RIV exhibited only 31.43% dissolution release after 60 min. After 1 h, study binary complexes prepared by kneading method and solvent evaporation method having 1:2 M ratio depicted 54.25% and 53.12% drug release respectively. This could be attributed to better wettability of molecule due to the companionship of cyclodextrin, which can bring down the interfacial tension between drug molecules and dissolution medium [28] This can also be credited to entrapment of pure drug molecule into the cavity of cyclodextrin. Ameliorated dissolution profiles as compared to parent drug are the characteristics of inclusion complexes [29].

The inclusion of various polymers improved the dissolution release of ternary complexes as well. It was found that ternary complex RIV: βCD: SOLO 10% (1:2: SOLO 10%) prepared by S.E method depicted superior results and ameliorated drug release of 78.13% after 90 min of study, while pure drug depicted only 33.23% release after the same interval. This could be due to the influence of two solubilizers (βCD and SOLO) in the ternary inclusion complex having a synergistic effect on RIV solubility [30]. Ternary complexes with PXM-188 depicted superior results than their binary complexes but less than PVP K-30 and SOLO. This can be due to the presence of clustered hydrates in the mixture, resulting in increment of thickness of the diffusion layer and eventually hampering the RIV release [31].

Results for dissolution effectiveness can be seen in Table 4. ANOVA was used to statistically confirm the DE findings. When the DE of binary inclusion mixtures was analyzed with the RIV, a statistically significant difference (p 0.05) was found. Ternary complexes also depicted enhanced DE and significant difference of (p < 0.05) when compared with binary mixtures. Keeping in view the all above data, it can be concluded that ternary complexes of SOLO prepared by SE method had improved the solubility and dissolution profile of RIV. This claim may be supported with less crystallinity of RIV as confirmed from DSC and XRD analysis.

**Table 4:** Dissolution parameters values for RIV, binary and ternary systems. Mean ± SD, n = 3.

RIV: βCD: POLYMER	DE60 (%)
RIV	19.03 ± 0.19
RIV: βCD KM (1:1)	38.24 ± 0.11
RIV: βCD KM (1:2)	43.77 ± 0.15
RIV: βCD KM (1:4)	41.91 ± 0.13
RIV: βCD SE (1:1)	31.85 ± 0.14
RIV: βCD SE (1:2)	42.09 ± 0.09
RIV: βCD SE (1:4)	39.84 ± 0.18
RIV: βCD: PXM-188 KM (1:2:2.5%)	45.46 ± 0.17
RIV: βCD: PXM-188 KM (1:2:5%)	46.39 ± 0.29
RIV: βCD: PXM-188 KM (1:2:10%)	47.67 ± 0.16
RIV: βCD: PXM-188 SE (1:2:2.5%)	48.35 ± 0.19
RIV: βCD: PXM-188 SE (1:2:5%)	49.08 ± 0.22
RIV: βCD: PXM-188 SE (1:2:10%)	49.63 ± 0.25
RIV: βCD: PVP K-30 KM (1:2:2.5%)	46.28 ± 0.14
RIV: βCD: PVP K-30 KM (1:2:5%)	48.60 ± 0.17
RIV: βCD:PVP K-30 KM (1:2:10%)	49.00 ± 0.14
RIV: βCD: PVP K-30 SE (1:2:2.5%)	48.55 ± 0.06
RIV: βCD: PVP K-30 SE (1:2:5%)	49.13 ± 0.19
RIV: βCD:PVP K-30 SE (1:2:10%)	51.99 ± 0.23
RIV: βCD:SOLO KM (1:2:2.5%)	46.03 ± 0.08
RIV: βCD:SOLO KM (1:2:5%)	48.37 ± 0.19
RIV: βCD: SOLO KM (1:2:10%)	50.41 ± 0.01
RIV: βCD: SOLO SE (1:2:2.5%)	53.95 ± 0.14
1:2:5% (RIV: βCD:SOLO SE (1:2:5%)	56.03 ± 0.32
1:2:10% (RIV: βCD: SOLO SE (1:2:10%)	57.01 ± 0.12

### 3.4 Fourier transform infrared spectroscopy (FTIR*)*

**Fig. 4:**
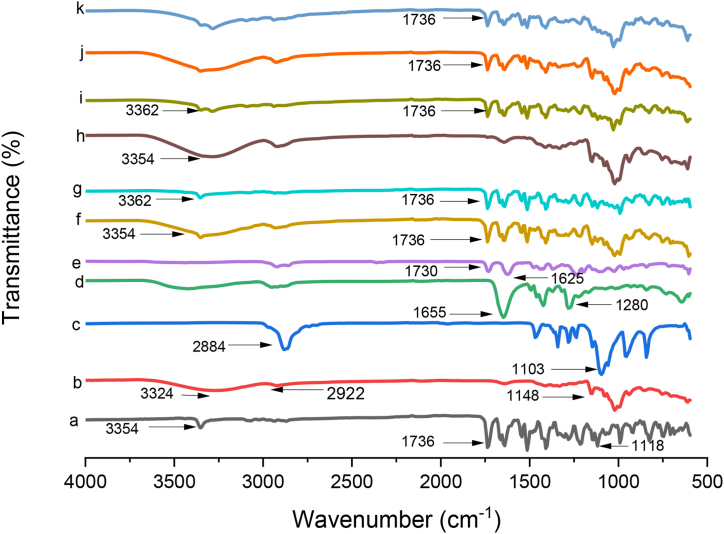
FTIR Spectrum of (a) RIV, (b) βCD, (c) PXM-188 (d) PVP K-30 (e) SOLO (f) RIV: βCD K.M (1:2) (g) RIV: βCD S.E (1:2) (h) RIV: βCD: PXM-188 SE (1:2:10%) (i) RIV: βCD: PVP K-30 SE (1:2:10%) (j) RIV: βCD: SOLO KM (1:2:10%) and (k) RIV: βCD: SOLO SE (1:2:10%).

The interaction of RIV with βCD can be investigated with spectroscopic technique. When different components are blended at the molecular level, the oscillating dipole of the molecule's changes. These variations can be seen in the frequency, bandwidth, and strength of the interacting groups. The FTIR spectrum of pure RIV depicted characteristics peaks of C–O ether stretch at 1118 cm^−1^, C=O ester bond at 1736 cm^−1^ and secondary amine C–N stretch at 3354 cm^−1^ Fig. 4(a). The spectrograph of βCD depicted characteristics peaks of vibrational stretching and bending of O–H group, aliphatic C–H group and C–C bonds at 3324 cm^−1^, 2922 cm^−1^ and 1148 cm^−1^respectively Fig. 4(b). PXM-188 depicted absorption peak at 2884 cm^−1^(C–H stretch aliphatic), 1312 cm^−1^ (in plane O–H bend) and 1110 cm^−1^ due to (C–O stretch) [32]. PVP K30 depicted distinct absorption peak at 1625 cm^−1^, which could be attributed to carbonyl group. The broad spectrum at 3570 cm^−1^ were visible which might be due to the O–H stretching vibrations of the absorbed water molecules Fig. 4(c). PVP K30 indicated absorption peak at 1280 cm^−1^, which belonged to the O–H in-plane bending vibration Fig. 4(d) [33]. In the spectrum of SOLO Fig. 4(e), peaks detected at 1729.9 cm^−1^ and 1624.8 cm^−1^ were due to the carbonyl groups of the ester and caprolactam ring [34].

The peaks of RIV in the region of 1800–800 cm^−1^ and 3354 cm^−1^ were found in all the binary inclusion complexes prepared by kneading Fig. 4(f) and solvent evaporation Fig. 4(g) method at the same wavelength, but the intensity reduced and no new peaks were detected due to the dilution effect. The characteristics peak pattern of the RIV was not altered considerably, so it can be concluded that RIV had no chemical interactions with βCD in binary formulations [35].

In case of RIV:βCD: PXM-188 (1:2:10%) Fig. 4(h) prepared by solvent evaporation method characteristics peaks at 3354 cm^−1^ was present in the complex but with diminished intensity and the peak at 1736 cm^−1^ got disappeared which might be due to involvement of carboxylic functional group in the inclusion complexation [36]. The FTIR spectrum of RIV: βCD: PVP K-30 10% as shown in Fig. 4(i), depicted changes in characteristic peaks of RIV. Furthermore, certain RIV peaks are lost in the FTIR spectrum of the PVP K-30 ternary complex. The modification or disappearance of RIV's distinctive peaks could be attributed to RIV entrapment into CD cavity [37].

In case of soluplus, the characteristics peak of RIV disappeared at wavelength 3354 cm^−1^ in both ternary mixtures prepared by kneaded Fig. 4(j) and solvent evaporation Fig. 4(k) method. These results could be explained by the formation of a hydrogen bond between the active moiety and SOLO. The stability of the formulations could be by the presence of hydrogen bonding between the drug and the polymer [38].

#### 3.4.1 Scanning electron microscopy (SEM)

**Fig. 5:**
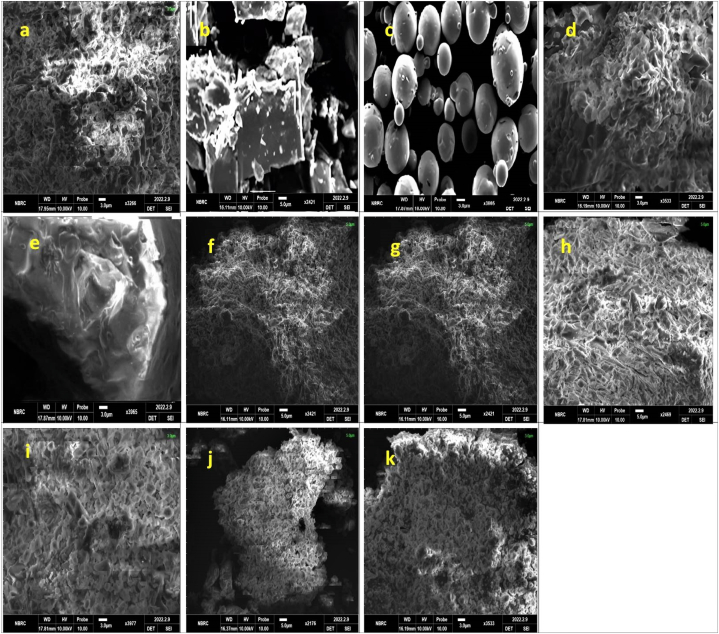
SEM of (a) RIV, (b) βCD, (c) PXM-188, (d) PVP K-30, (e) SOLO,(f) RIV: βCD K.M(1:2) (g) RIV: βCD S.E (1:2) (h) RIV: βCD: PXM-188 SE (1:2:10%) (i) RIV: βCD: PVP K-30 SE (1:2:10%) (j) RIV: βCD: SOLO KM (1:2:10%) and (k) RIV: βCD: SOLO SE (1:2:10%).

SEM is employed to investigate the surface morphology of optimized formulations. Photo images of pure RIV, βCD, PXM-188, PVP K-30, SOLO, binary and ternary inclusions are shown in Fig. 5. RIV consists of deposition of small and large particles on the surface as shown in Fig. 5(a) βCD has rough and irregular outer surface with rhomboidal crystals as shown in Fig. 5(b). RIV: βCD (1:2)complex prepared by kneading method in Fig. 5(f) depicted rough structure and RIV was entrapped in βCD cavity with changes of βCD from spherical to flat shape [39]. On the other hand, RIV: βCD (1:2) binary complex synthesized with solvent evaporation method Fig. 5(g) had more denser and fused particles on the surface indicating less crystallinity. RIV/βCD emerged as loose aggregates of irregularly scattered particles with a heterogeneous distribution. It can be concluded that binary complexes revealed less crystallinity than pure RIV in both preparation methods.

PXM-188 showed fine globular shape as shown in Fig. 5(c). Ternary inclusion complex of PXM-188 were observed in aggregated form having small particles Fig. 5(h). PVP K30 consisted of spheres with concave depressions as shown in Fig. 5(d). RIV was dispersed and disappeared in PVP K 30 ternary complex, confirming the change in crystallinity of the pure drugs Fig. 5(i) [40]. SOLO had revealed spherical particles dispersed on rough surface Fig. 5(e). Ternary complex RIV: βCD: PXM 188 (1:2:10%) had more amorphous nature Fig. 5(h) than binary complexes. RIV: βCD: SOLO (1:2:10%) showed regular and small particles on smooth surface and amorphous nature Fig. 5(j and k). This may be due to the entrapment of complete drug in cavity of cyclodextrin and amphiphilic nature of SOLO [41]. Hence method of preparation, entrapment within cyclodextrin cavity and nature of polymer were instrumental in transformation of crystal structure of RIV into amorphous one [42].

### 3.5 X-ray diffractometry (XRD)

**Fig. 6:**
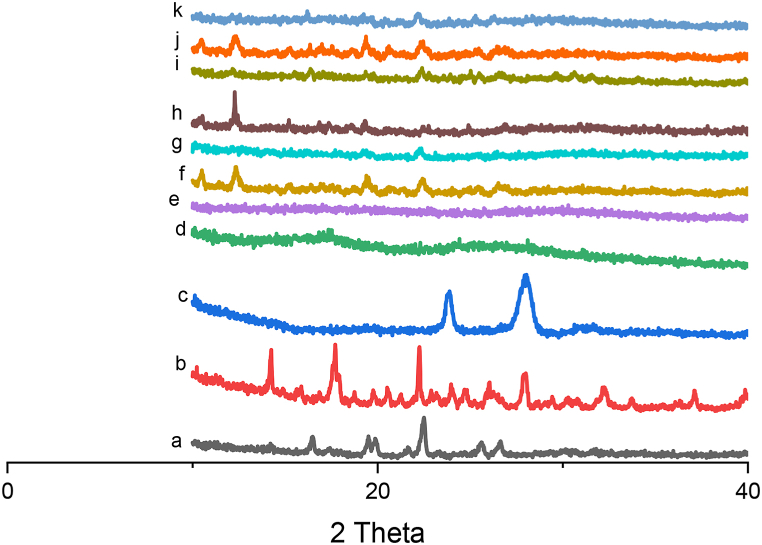
XRD Diffractograms of (a) RIV, (b) βCD, (c) PXM-188, (d) PVP K-30, (e) SOLO(f) RIV: βCD K.M(1:2) (g) RIV: βCD S.E (1:2) (h) RIV: βCD: PXM-188 SE (1:2:10%) (i) RIV: βCD: PVP K-30 SE (1:2:10%) (j) RIV: βCD: SOLO KM (1:2:10%) and (k) RIV: βCD: SOLO SE (1:2:10%).

Diffractogram of the RIV exhibited crystalline structure, having characteristics peaks at 2θ value of 22° Fig. 6(a). Diffractogram of βCD showed sharp peaks at diffraction angles (2θ) 14.1°, 17.7° and 22.2° showing a typical crystalline pattern Fig. 6(b). PXM-188 Fig. 6(c) depicted the characteristics peaks at 2θ value of 24^°^ and 26^°^ due to its crystalline structure Whereas PVP-K30 Fig. 6(d) and soluplus Fig. 6(e) exhibited no such peaks which is representative of its amorphous form. The RIV-βCD inclusion complexes in Fig. 6(f) depicted reduced intensity peaks as compare to pure drug. The kneading method could influence crystal structure in the inclusion powder. This could be attributed to the dilution of drug in the cavity of cyclodextrin. RIV: βCD SE (1:2) showed less crystalline appearance with reduced intensity RIV peaks Fig. 6(g). The promotion of amorphousness resulted in ameliorated dissolution and solubility [43].

Diffractogram images of ternary complexes manifested less crystalline nature as compared to RIV and binary complexes. For the ternary system with βCD/PXM-188, the characteristic peak of PXM-188 diminished and almost flat region was observed in Fig. 6(h). For the ternary system with βCD/PVP K-30 characteristics peak of drug was diminished significantly Fig. 6(i). Ternary system with SOLO Fig. 6(j and k) showed merging or loss of characteristic peaks of RIV compared to other ternary complex hinting at complete entrapment of RIV into βCD cavity. The XRD plots showed the typical crystalline peaks of RIV (with less number and reduced intensity). Therefore, it can be concluded that presence of little crystallinity of RIV was observed in the binary inclusion complexes but final ternary formulations appear to be more amorphous in nature.

### 3.6 Differential scanning calorimetry (DSC)

**Fig. 7:**
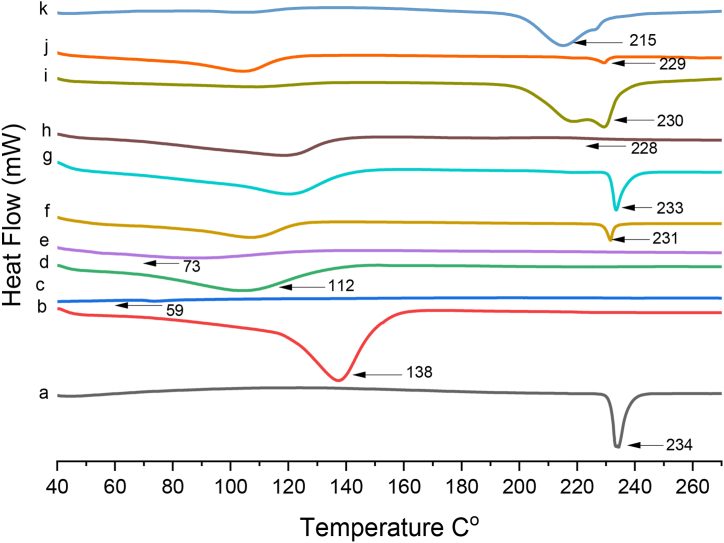
Thermograms of (a) RIV, (b) βCD, (c) PXM-188, (d) PVP K-30, (e) SOLO,(f) RIV: βCD KM (1:2) (g) RIV: βCD SE (1:2) (h) RIV: βCD: PXM-188 SE (1:2:10%) (i) RIV: βCD: PVP K-30 SE (1:2:10%), (j) RIV: βCD: SOLO KM (1:2:10%) and (k) RIV: βCD: SOLO SE (1:2:10%).

DSC is a thermo analytical technique employed to evaluate the phase transitions of free drug (RIV), βCD, (PXM-188, PVP K-30 and SOLO), binary and ternary inclusion complexes prepared by different methods. In DSC thermogram, RIV exhibited a sharp endothermic peak at 234 °C Fig. 7(a) which conformed with its melting point [44]. βCD indicated broad endothermic peak at 138 °C in Fig. 7(b) which might be due to exsiccation. PXM-188 and PVP K-30 showed endothermic peak at 57 °C and 101 °C respectively (Fig. 7(c) and (d). Binary complexes (RIV: βCD (1:2)) prepared by kneading method depicted reduced intensity of endothermic peaks and shift of peak from 234 °C to 231 °C which might be credited to the partial entrapment of guest molecule in βCD cavity Fig. 7(f). RIV: βCD (1:2) binary complex prepared by solvent evaporation method depicted reduced intensity of endothermic peak which can be anticipated as a result of complexation Fig. 7(g). Hence it can be concluded that binary complexes prepared by kneading and solvent evaporation method depicted pronounce shift of peak and reduction in intensities of endothermic peaks [45].

PXM-188 10% Fig. 7(h) exhibited a semi flat region and partial absence of peak on the thermogram hinting drug amorphization and optimum interaction with βCD [46]. Ternary complex RIV: βCD: PVP K-30 (1:2:10%) manifested clear shift change to 230 °C and diminished intensity peak indicating RIV less crystallinity Fig. 7(i).

SOLO thermogram is shown in Fig. 7(e). Thermogram of 1RIV: βCD: SOLO (1:2:10%) Fig. 7 (j) prepared by kneading method depicted significant reduction in endothermic peak strength and semi flat region of RIV. DSC of RIV: βCD: SOLO SE (1:2: 10%) manifested obvious change in shift to 215 °C and loss of crystallinity of RIV Fig. 7(k). This was due to the presence of SOLO and complexation with the cyclodextrin in ternary complexes. The solvent evaporation method outperformed the kneading method in the production of ternary complexes [36]. Hence, it can be concluded that ternary complexes prepared with SOLO by solvent evaporation method exhibited nature that is more amorphous and shift of endothermic peak as compare to other ternary complexes.

## 4 Conclusions

Binary inclusion complexes of RIV: βCD were prepared successfully. The inclusion complex of RIV:βCD (1:2) produced by kneading and solvent evaporation technique improved RIV solubility and dissolution profile. The solid-state characterization results showed that the complex formed with a highly agglomerated and amorphous RIV structure. The addition of polymers (PXM 188, PVP K-30 and SOLO) in different molar concentrations further enhanced the solubility and drug release. Characterization studies also confirmed the formation of new complexes and amorphous nature of RIV. Hence ternary complexes with soloplus (SOLO) prepared by solvent evaporation method showed superior results and increment in solubility and dissolution profile of RIV.

## Author contribution statement

Waqas Haider Khan: Performed the experiments; Analyzed and interpreted the data; Wrote the paper. Sajid Asghar: Analyzed and interpreted the data. Ikram Ullah khan: Contributed reagents, materials, analysis tools or data. Abdulrahman Alshammari: Contributed reagents, materials, analysis tools or data. Muhammad Irfan: Contributed reagents, materials, analysis tools or data. Muhammad Shahid Riaz Rajoka: Contributed reagents, materials, analysis tools or data. Rabia Munir: Contributed reagents, materials, analysis tools or data. Pervaiz A Shah: Contributed reagents, materials, analysis tools or data. Ikrima Khalid: Contributed reagents, materials, analysis tools or data. Fizza Abdul Razzaq: Analyzed and interpreted the data. Syed Haroon Khalid: Conceived and designed the experiments; Analyzed and interpreted the data; Wrote the paper.

## Data availability statement

Data is available on request.

## Declaration of competing interest

The authors declare that they have no known competing financial interests or personal relationships that could have appeared to influence the work reported in this paper.
